# The Significance of Enlarged Lymph Nodes in Children

**Published:** 1911-12

**Authors:** George Kent Varden

**Affiliations:** Atlanta, Ga.


### THE SIGNIFICANCE OF ENLARGED LYMPH NODES IN CHILDREN.

By George Kent Varden, M. D., Atlanta, Ga.

In infancy and chilhood, and particularly during the former period, there is a marked tendency to lymphatic enlargement.

On account of its greater frequency we shall speak first of the chronic enlargement.

This is discovered many times in cases of faulty or difficult feeding, in that class of cases that is constituted largely by infantile atrophy, in which there are no gross errors of diet and yet there is not only failure to gain in weight, but a tendency to waste.

The superficial nodes are palpable in neck, inguinal region and axillae. Sometimes in only one locality and again in all. The nodes are small, firm, unattached to the overlying skin and rarely suppurate. At autopsy deep nodes are found enlarged in the same way. Nothing can be found other than a depraved state to account for the enlargement, yet we must think of the absence of thyroid secretion in artficial feeders as a possible explanation. This is suggested by the report that adenoids do not so often recur after removal in cases where thyroid extract is used after operation.

This enlargement is too often taken as indicating syphilis and while this disease might co-exist, I would not allow it to influence my judgment unless there was other evidence of more weight.

Conditions of the scalp, mouth, teeth and naso-pharynx are often responsible for cervical enlargements. Pediuli, eczemas, impetigo, and pityriasis capitis are examples of what may cause it in the neck posteriorly. An apthous or ulcerative stoamatitis may be found to cause the enlarged sub-maxillary group. In diseases of the naso-pharynx the retropharyngeal nodes may be involved, producing suppuration and with it a retro-pharyngeal abscess. Tonsillar disease and infection of the middle ear are more often followed by greater enlargment and at times suppurate. Enlargement just in front of the sterno-mastoid muscle simulates epidemic parotitis or mumps and when the sub-maxillary is involved it may be confused with mumps involving the submaxillary salivary gland. The infrequency of mumps in the very young, its shorter duration and the fact that suppuration is unusual, is against diagnosis of this infection. Hodgkin's disease is rare in infancy and when it does occur the enlargement is bilateral, one side is larger than the other and the size attained is much greater than in simple lymphatic enlargement. Glands in the abdomen may be felt and the anaemia is extreme.

Tenderness is absent and the cases usually end fatally.

Tuberculous adenitis is a disease that is not often seen before the third year of life, the growth is slow, breaking down with conseuentq scarring is frequent and pain is not often complained of by patient on examination. Adenitis limited to the inguinal group is met in cases of vulvo-vaginitis and in gonorrhoeal urethritis in the young male. As in older individuals when the enlargement is single and of any size it may be confounded with hernia.

In children whose ages vary between four and twelve years there occurs a disease first described by West as Glandular Fever, characterized by adenopathy, anorexia, variable temperature and duration of about two weeks. The glands involved are more often superficial and the cervical, axillary and inguinals may all be increased in size or as sometimes happens, only a single group. When the neck group is involved, the clinical picture may suggest a simple torti collis or disease of the cervical vertebrae. An epidemic is the rule though isolated cases occur. There seems a tendency for this to spring up in institutions. Suppuration is rare and recovery is the rule.

I have intentionally omitted mentioning the glandular involvement that occurs in such infectious disease as scarlet fever, measles, German measles, diphtheria and pertussis as you all are too wel acquainted with these conditions.

Lymphatic leukaemima, and in fact, all leukaemias occur very rarely before the sixh year and are to be recognized by the blood picture, including a red and white cell count along with a differential blood count.

The susceptibility of the lymph structures in early life to hyperplasia with or without suppuration is much greater than in the adult, and remains so until after puberty. Just what part the thymus, thyroid and para-thyroids play in this connection is at present little understood. The fact that exposure of the enlarged thymus to X-Ray is indicative of a co-relation.

In closing, it may be fitting to say a word concerning the treatment of the more prevalent forms of adenitis.

For the chronic nodes of atrophic infants well regulated feeding is the only thing that will be attended with result and even then this result comes slowly. Local applications are unnecessary and of no value.

In syphilitic cases constitutional treatment plus judicious feeding is all that is necessary. The last is extremely important in congenital cases.

In tubercular cases tuberculin given subcutaneously in very minute doses without constitutional reaction often causes disappearance. If broken down and suppurating pus should be evacuated and this followed by tuberculin.

For those cases of simple adenitis where there is a tendency to suppuration much can be done by local application. Iodin, which is used a good deal, is apt to produce a dermatitis and accomplishes little. Ichthyol ointment (20-25%) applied freely on starch free cloth meets requirements better than anything I have used. Belladonna ointment may be incorporated with the ichthyol. How ichthyol acts in glandular affections I am unprepared to state, yet I am convinced that it is of decided value in certain cases. Syrup of the iodide of iron has many supporters as an internal remedy. It has two objections that are sufficient to make its use limited, the fact that it is irritating to the young when all right, and the free iodine in many of the preparations makes it more irritating. Whether it has more than tonic effect is open to question.

When suppuration is inevitable moist heat in the form of poultices hastens suppuration and relieves pain. After suppuration has taken place surgical interventon is required.

Candler Building, Atlanta, Ga.

